# “This can certainly work…”: stakeholder perspectives of the feasibility of a caregiver-led training program for caregivers of children with cerebral palsy in a rural setting in Malawi

**DOI:** 10.3389/fpubh.2024.1390645

**Published:** 2024-07-03

**Authors:** Takondwa Connis Bakuwa, Gillian Saloojee, Wiedaad Slemming

**Affiliations:** ^1^Department of Rehabilitation Sciences, Kamuzu University of Health Sciences, Blantyre, Malawi; ^2^Division of Community Pediatrics, Department of Pediatrics and Child Health, Faculty of Health Sciences, University of Witwatersrand, Johannesburg, South Africa; ^3^Department of Physiotherapy, Faculty of Health Sciences, University of Witwatersrand, Johannesburg, South Africa; ^4^Children’s Institute, Department of Pediatrics and Child Health, Faculty of Health Sciences, University of Cape Town, Johannesburg, South Africa

**Keywords:** implementation, caregiver-led, training, program, cerebral palsy, feasibility, Malawi

## Abstract

**Introduction:**

Caregiver training is a key component of rehabilitation for children with complex lifelong disabilities such as cerebral palsy. However critical shortages of therapists in low- and middle-income countries like Malawi, reduce access to therapy. Introducing expert caregivers to assist with the provision of basic training on the condition for fellow caregivers offers a potential solution. However, there is a paucity of evidence regarding the implementation of such strategies in low-resource settings. The aim of this study was to explore perspectives of stakeholders regarding the feasibility of implementing a caregiver-led and delivered training program for caregivers of children with cerebral palsy in Malawi.

**Methods:**

Over 5 days in January 2023, a caregiver-led training program, the “Malamulele Onward Carer-to-Carer Training Program,” was conducted in Blantyre, Malawi. A South African master trainer traveled to Malawi and delivered the program to potential stakeholders including caregivers of children with cerebral palsy; physiotherapists; and community-based organization representatives. Stakeholder perspectives regarding the acceptability, demand, practicality and adaptation of the program were obtained through a combination of focus group discussions, in-depth interviews, and daily field notes. Data from the focus group discussions and in-depth interviews were analyzed using thematic analysis.

**Results:**

The caregiver-led training program was deemed acceptable despite two areas identified as potential areas of concern; that the expert caregivers may cross practice boundaries and that their fellow caregivers may look down upon them. A demand for this program was expressed because of perceived relative advantages and relevance to caregiver needs. Participants indicated that the intervention could be easily delivered using local materials, absorbed and supported by existing community structures.

**Conclusion:**

A caregiver-led training program offers an innovative way of supporting caregivers of children with complex disabilities such as cerebral palsy in low-resource settings. The stakeholder engagement demonstrated the positive perspectives of all stakeholders. The areas for modification and adaptation highlighted by the stakeholders will be useful in strengthening the implementation of the program in Malawi.

## 1 Introduction

Cerebral Palsy (CP) is a complex developmental movement disorder that presents with a variety of associated impairments, including epilepsy and cognitive difficulties (1). These disorders result in limited participation in activities of daily living (2). As a consequence of the lack of early therapeutic intervention and comprehensive lifelong rehabilitation, CP continues to cause severe disability in children in low- and middle-income countries (LMICs) (3). Currently, the global prevalence of CP ranges between 1.5 to 4 per 1,000 children (3, 4). The highest numbers of children with severe CP are in the rural areas in LMICs (5) leading to a significant health burden in these settings.

A population-based study conducted in two rural settings in Malawi reported CP as the commonest cause of motor disability, accounting for a quarter of all children with physical impairment (6). The study estimated that 2.1 children per thousand would benefit from physiotherapy services. However, access to early intervention and a continuum of care for children with CP is a challenge in Malawi with only 0.8 physiotherapists per 100,000 people; the majority of whom work in the four-government urban-based tertiary hospitals. A critical scarcity of rehabilitation services for children with CP, especially in rural areas therefore exists.

The complexity of CP requires frequent, regular and long-term therapeutic input to reduce disability (7). The caregiver needs to be knowledgeable about the specific type of CP their child has to ensure proper tailoring of therapies and adjustment of activities of daily living. For example, mobility, positioning and considerations for supportive devices vary depending on the type of CP the child has, its severity and the respective motor functioning levels (7, 8). In addition to this, the prognosis of the child’s condition also varies and that knowledge is important for managing the caregiver’s expectations, well-being and outlook on care needs (8). Moreover, children with CP vary in their participation abilities including play and can be challenged by the existence of other associated impairments including cerebral visual impairment (2). Provision of basic caregiver skills training in these aspects has been found to improve caregiver knowledge, skill and well-being and also functional outcomes of their children with CP (9, 10). It is the role of rehabilitation professionals to provide targeted caregiver training in all these aspects, however, workforce shortages in LMICs reduce their capacity to adequately train caregivers.

In line with the task-sharing strategy proposed by the World Health Organization (WHO), caregiver-mediated interventions have been recommended as an effective approach to harness the agency of caregivers to improve access to practical knowledge and skills in LMICs (11–13). These approaches focus on the engagement of primary caregivers of children with a disability, usually parents, and are commonly referred to as “parent-mediated/delivered” interventions (14). These programs are increasingly called “caregiver-led/delivered” approaches; which acknowledge that the child’s primary caregiver may not always be a parent but could also be grandparents, aunts, uncles and siblings (15). In 2019, the WHO Caregiver Skills Training (CST) program was developed to support training and well-rounded support for caregivers of children with neurodevelopmental disabilities, specifically Autism, in LMICs (16). The WHO CST has been successfully adapted and implemented in several LMICs and is designed to be deliverable by non-specialists including “peer-caregivers” to enhance reach and scale up (17, 18). During the recent implementation of the WHO CST, these non-specialists have included community health workers, teachers and primary health workers. However, the engagement of caregivers themselves as program facilitators of the WHO CST is yet to be documented.

A South African non-profit organization, Malamulele Onward, championed the training of caregivers to work as peer facilitators (referred to as “expert caregivers” in this paper). In consultation with caregivers, the organization developed a training package, the Malamulele Onward Carer-2-Carer Training Program (MOC2CTP). Expert caregivers are trained to deliver a series of seven workshops to other caregivers of children with CP. The seven workshops impart practical knowledge and skills to caregivers which assists caregivers in understanding their child and the nature of CP. The workshops focus on teaching helpful caregiving practices which are integrated into daily life to enable each child to reach their potential while decreasing the burden of care. Over the past 7 years, the organization has trained over 50 expert caregivers in rural South Africa, Lesotho, and most recently Uganda (19). Qualitative studies involving over 400 caregivers who attended the program have demonstrated that the MOC2CTP results in increased caregiver understanding of both CP and their child; decreased feelings of isolation and self-blame; and feelings of hope and confidence in caregiving (20).

While caregiver-led and delivered training programs are being advocated and used for caregivers of children with various neurological conditions, including Autism and Zika-related congenital disorders (21, 22) there is limited work being done to formally study the feasibility of this approach specifically for caregivers of children with CP. Feasibility relates to the possibility of implementing an intervention or a program in a new setting. It involves assessing factors such as acceptability, appropriateness, relevance and practicality in terms of the availability of resources and time (23, 24). This means that considerations may vary depending on the specific end users of the intervention or setting of intervention. Therefore, establishing feasibility is necessary to ensure that a program is well-prepared for application within a particular practice setting to yield the intended results (24).

Recent feasibility studies that included caregiver-delivered aspects and also potentially children with CP have only focused on children at risk of neurodevelopmental conditions who were younger than 2 years (12, 13) and hence may not be relevant for older children with CP. There is a gap in the evidence regarding the feasibility of caregiver-led and delivered programs for children with CP. Moreover, in a setting like Malawi, where rehabilitation services are mainly led and delivered by physiotherapists, it is not known if this approach would be deemed acceptable, practical or adaptable to a local context. Therefore, this study sought to explore stakeholder perspectives regarding the feasibility of a caregiver-led training program, specifically the MOC2CTP for caregivers of children with CP in Malawi. Stakeholders refer to the end users; which are the caregivers and all the groups of people vested in the successful delivery of the program (25, 26); in this case, physiotherapists and the community based organization working with the caregivers.

## 2 Materials and methods

### 2.1 Study design

This was a qualitative study, using focus group discussions (FGD) and in-depth interviews (IDI) to elicit stakeholder perspectives. It comprises the first phase of a larger pilot implementation study.

The study design and tools were informed by Bowen’s feasibility framework, which describes eight constructs for studying feasibility, *viz.*, acceptability, demand, practicality, adaptation, implementation, limited efficacy, integration and expansion (23). This study focused on four of these constructs: acceptability, demand, practicality and adaptation, as these were deemed relevant to guide the subsequent pilot implementation. The development of the data collection tools was informed by the Consolidated Framework for Implementation Research (CFIR) (27, 28) which provides tailored interview and focus group topic templates related to aspects of acceptability, demand, practicality and adaptation among other feasibility constructs (Table 1).

**Table 1:** Feasibility constructs informing the study interview and topic guides.

Construct	Description according to Bowen’s Framework (23)	CFIR guide tools (27)	Example questions derived from CFIR tools
Acceptability	The reaction of participants and stakeholders to the training program & concept of a peer facilitator	Intervention characteristics: complexity (CIFR) Inner setting (implementation climate) (CIFR)	How complicated is the session/intervention? What is the general level of receptivity in your organization to implementing the intervention?
Demand	The perceived extent to which the new MOC2CTP is likely to be deemed necessary, utilized and received	Outer setting: patient needs (CIFR) Intervention characteristics: relative advantage (CIFR)	How well do you think the intervention will meet the needs of the individuals served by your organization? How does the intervention compare to other similar existing programs in your setting?
Practicality	The extent to which the MOC2CTP is delivered with the limited means, resources and circumstances of the context	Intervention characteristics: Design and packaging, cost (CIFR) Implementation readiness: self-efficacy (CIFR)	What is your perception of the quality of the supporting materials, packaging, and bundling of the intervention for implementation? How confident are you that you will be able to successfully implement the intervention?
Adaptation	Changes in the MOC2CTP that may have to be made for the intervention to accommodate contextual factors unique to Namwera	Intervention characteristics: Adaptability (CFIR) Inner setting: structural characteristics (CIFR)	What kinds of changes or alterations do you think you will need to make to this session/ intervention so it will work effectively in your setting?

### 2.2 Study setting

The study was conducted in January 2023, at the Feed the Children rehabilitation center in Blantyre, Malawi. The rehabilitation center was well situated for hosting this initial study to accommodate the visit of the master trainer from Malamulele Onward who would introduce and conduct the caregiver-led training program.

Participants included members of the Tiyende Pamodzi Group; a community-based organization (CBO) in the rural community of Namwera in the Mangochi district, situated in the southern region of Malawi. Tiyende Pamodzi is a local non-profit organization which provides monthly one-on-one physiotherapy, access to assistive devices and nutrition support for children with disabilities. In January 2023, the center had over 400 children with physical impairments registered, with CP being the most common condition. The organization employs one physiotherapist to serve the 12 subsidiary centers within their catchment area.

Tiyende Pamodzi works with the Mangochi District Hospital, which like most of the rural district hospitals in Malawi, has a shortage of physiotherapists and rehabilitation assistants and consequently the reach of services for areas as remote as Namwera is limited. In 2023, the district hospital had one physiotherapist and four rehabilitation technicians servicing a population of about 1,323,159 (29). This setting therefore depicts a typical resource-constrained rural area in Malawi, yet with a well-organized community structure that can be a conduit for introducing successful innovation of a caregiver-led intervention (30).

### 2.3 Study participants

Participants comprised stakeholders of the proposed caregiver-led and delivered training program. They included 10 caregivers of children with CP, two physiotherapists, two executive committee members from the Tiyende Pamodzi Group CBO who were purposively selected. The investigator (TB) organized a meeting with the executive committee of the Tiyende Pamodzi to present the concept of the study. In consultation with Tiyende Pamodzi’s physiotherapist, the executive committee chose the 10 caregivers; one from each of the 10 subcenters of the Tiyende Pamodzi group which would be involved during the implementation of the next phase of the study. Criteria for identifying caregivers were agreed upon by the research team and Tiyende Pamodzi executive committee. These criteria included being able to read and write, having at least 2 years of experience in primary caregiving of a child with CP and at least 1 year of involvement with the CBO, having a similar socioeconomic status as the majority of the caregivers, and having displayed qualities such as initiative, leadership, and empathy during community center programs. These attributes were necessary as it was important to start identifying caregivers who would later go on to be trained *expert caregivers*. Similar studies conducted in other LMICs have identified these traits as important for *expert caregivers* or *peer supporters* (20, 21).

The two executive committee members were selected based on their experience working with the CBO and their knowledge of its visions and goals. They also needed to have experience with the field work done by the CBO, empathy and a keen interest in learning. The two physiotherapists were selected based on their work experience with caregivers of children with CP, their interest in improving service delivery in rehabilitation care, and their willingness to participate in the subsequent implementation study. The study also gathered demographic details of the children with CP, including the type of CP and their level of functioning, using the Gross Motor Functioning Classification System (GMFCS). The GMFCS is a classification system that assesses the movement abilities and limitations of children with CP, including the need for assistive technology and wheeled mobility (1).

### 2.4 The training program

The Malamule Onward Carer-to-Carer Training Program (MOC2CTP) is a targeted skills training program which focuses on teaching caregivers to understand what CP is, identify the different types of CP, learn to problem-solving, positioning and handling skills related to each type of CP in the following areas; eating and drinking, play, communication and cerebral visual impairment. These aspects are embedded in blending therapy with activities of daily living and looking at CP management as a way of life. The main outcome of the program is improvement in caregiver knowledge and skills related to child positioning, feeding, mobilization, play and engagement, communication and vision. This is expected to improve child-level outcomes; including child-mobility, self-care, feeding, and social skills. The program also aims at improving caregiver well-being and quality of life.

The MOC2CTP was developed in collaboration with caregivers and designed to be delivered by caregivers of children with CP (*expert caregivers*) to fellow caregivers of children with CP in resource-constrained settings. It comprises seven workshops, each lasting between 2 and 2.5 h. Each session includes an ice-breaker activity based on themes of psychosocial support; information on a particular topic; a larger section of practical experiential demonstrations and as well as group discussions. Table 2 provides an outline of the content covered in the MOC2CTP.

**Table 2:** Content of the Malamulele Onward Carer-to-Carer program.

Module	Content
1	Introduction to CP: What is it and how does it affect my child
2	CP as a way of life: Looking after my child throughout the day
3	Getting active: Getting my child’s body ready to move throughout the day
4	Eating and drinking: making meal times safe and comfortable for my child
5	Communication: My child and I understand each other
6	Cerebral Visual Impairment: Understanding where and what my child can see
7	Play: Unlocking my child’s potential

### 2.5 Study procedures

A master trainer from Malamulele Onward in South Africa traveled to Malawi and delivered the seven-module training program to the study participants over 5 days. The master trainer is an experienced mother of a child with CP, who was initially trained to be a trainer by therapists at Malamulele Onward. She has over 7 years of experience in training peer facilitators in South Africa and also assisted in training expert caregivers in other countries including Uganda.

Drawing from Cornwall’s´ participation model (31) the three groups of participants in this study participated at two levels to strengthen the assessment of the program; program recipient level and program observant level. The caregivers were the active recipients of the intervention, while the therapists and CBO representatives played the role of professional observers with some active participation as required. This allowed for co-learning and co-reflection about the training program.

### 2.6 Data collection

Data were collected concurrently with the training program through focus group discussions (FGDs) and In-depth Interviews (IDIs). FGDs were conducted with the caregivers daily after each module was presented while IDIs were conducted with the physiotherapists and CBO representatives on the final day. It was necessary to conduct FGDs daily to ensure that caregivers recalled and shared module-specific perspectives regarding feasibility and in addition to the overall appraisal of the whole program which was conducted on the final day.

The primary author (TB) facilitated all FGDs and conducted all IDIs. A trained research assistant, a human and environmental health specialist (BSc) with 2 years of experience in qualitative data collection, was responsible for note-taking during the FGD and IDI. The research assistant also took notes during the debriefing session conducted by the master trainer on the final day. The focus of the debriefing session was on providing feedback related to the delivery of the program to the Malamulele Onward program developers. This was different from the focus of the FDGs and IDIs whose focus was exploring aspects related to the feasibility of the program in the new context.

#### 2.6.1 Focus group discussions

The daily FGDs were audio recorded and key points were captured and populated on a flip chart as the feedback continued to build each day. On the final day, the flipchart paper was displayed on the wall to prompt a reflective discussion with the caregivers regarding the program as a whole. The FGDs with the caregivers were conducted entirely in the local language, Chichewa. The daily post-session FGD with caregivers, took an average of 30 min while the FGD conducted on the final day lasted approximately 1 h.

#### 2.6.2 In-depth interviews

Two IDIs were conducted with the physiotherapists and CBO representatives on the last day of the five-day training program. The in-depth interviews were conducted in the form of homogenous dyadic interviews. This is a process whereby the two therapists were interviewed together, and the two CBO representatives were interviewed together. This approach ensured a co-construction of feedback and ideas, tapping from both their shared and unique perspectives about the study setting (32). The interviews were conducted in Chichewa; however, the therapists were free to express their responses in English. Thus, the data had a mixture of English and Chichewa. The dyadic interviews lasted between 45 and 60 min each.

Due to the pre-determined sample size for the study, cross-triangulation of perspectives between the three groups of participants was used was used to further explore new phenomena until conceptual maturity was reached (26, 27). Cross-triangulation was done on the last day and issues raised during the week by caregivers were cross-checked with the professional observers and rediscussed by caregivers in the last overall FGD (33, 34). The topic guides for the FGDs and IDIs contained similar questions specifically related to the acceptability, demand, practicality and adaptation of the intervention (Table 1). Prompts were added to guide the administrator and enhance the depth of responses.

### 2.7 Reflexivity

All three authors are physiotherapists. TB has a little over 5 years of work experience with children with CP and it was her first time working with the MOC2CTP. She was primarily involved in data collection as the principal investigator for the study. She had no prior relationship with the caregivers and CBO representatives involved in the study but had interacted with both physiotherapists involved in the study during undergraduate training and clinical internship program, respectively. WS and GS have extensive experience in working with children with disabilities, including CP, and have conducted research in the field. GS led the development of the MOC2CTP and has over the years sought to understand how the program would work in other local and regional settings.

The authors reflected on their prior understanding throughout the research processes and were mindful to let the analysis be data-driven. Their experience enriched the depth of analysis and reflective enquiry.

### 2.8 Data management and analysis

The audio-recorded data were transcribed verbatim, in the original language which was mostly Chichewa (and some English) using Microsoft Word. The data were then translated into English and imported into NVivo software for management and analysis.

The analysis of the data was theoretically driven by Bowen’s feasibility framework (23). Four constructs from this feasibility framework were used as global priori deductive thematic areas. Constructs from the CFIR were also used to describe organizing themes. An iterative process was followed during data analysis to derive preliminary emerging impressions from the data. This was followed by systematic coding of the data, using both inductive and deductive analysis. The data were initially analyzed inductively, where codes were generated as the researcher read through all sections of the data line-by-line assigning codes to segments of the data as they unfolded. These codes then underwent a second level coding whereby the original codes were reanalyzed deductively through categorization into basic themes that fed into the four pre-selected concepts of Bowen’s feasibility framework (35, 36). Co-investigators checked for quality and agreement in codes.

### 2.9 Ethical considerations

Ethical approval was obtained from the Human Research Ethics Committee (Medical) at the University of Witwatersrand in South Africa (M220924) and the College of Medicine Research Ethics Committee at the Kamuzu University of Health Sciences in Malawi (P.04/22/3608). Written informed consent was obtained from all participants.

## 3 Results

Ten caregivers, two physiotherapists and two representatives from the community-based organization participated in the study. The community and district-level physiotherapists had approximately 2 years of experience working in the community and the district hospital, respectively. The CBO representatives had over 5 years of experience working with the CBO and working with children with disabilities and their families in the community. Table 3 describes the 10 caregivers who participated. Characteristics of their children are also described, including the child’s level of movement function (denoted by “GMFCS”), and the type of CP the child presented with.

**Table 3:** Description of the study sample.

ID	Caregiver			Child			
	Age	Relationship	Marital status	Age	Sex	GMFCS	CP subtype
Caregiver01	25	Mother	Single	6	Girl	4	Choreo-athetoid
Caregiver02	37	Mother	Divorced	3	Girl	5	Spastic
Caregiver03	43	Father	Married	5	Boy	4	Spastic
Caregiver04	28	Mother	Single	12	Boy	3	Spastic
Caregiver 05	24	Mother	Married	2	Boy	3	Spastic
Caregiver 06	38	Grandmother	Divorced	7	Female	5	Spastic
Caregiver 07	42	Mother	Widowed	15	Female	2	Mixed
Caregiver08	42	Aunt	Widowed	6	Female	4	Choreo-Athetoid
Caregiver09	37	Mother	Married	4	Male	5	Dystonic
Caregiver10	35	Mother	Divorced	2	Male	4	Dystonic

### 3.1 Qualitative findings

Several subthemes were identified under each of the priori deductive global themes (implementation construct), i.e., acceptability, demand, practicality and adaptability. Table 4 summarizes the basic themes arising from the data.

**Table 4:** Summary of themes identified for each thematic area.

Implementation construct	Organizing theme	Basic themes
Acceptability	Intervention characteristics (Advantages)	Knowledge of my child
		The program was well-organized
		Value in being taught by fellow caregivers
		Lots of learning
	Intervention characteristics (Potential disadvantages)	Caregivers looking down on the peer facilitator
		Caregiver facilitators not knowing their limits
	Implementation climate (positive)	Aligned with CBO goals
		Support structures available
Demand	Relative advantage	Complement to existing interventions
		Less burdensome to caregivers
	Patient needs met by program	Learning how to deal with practical issues faced
		Acquiring new knowledge
	Patient needs not met by program	Need to address toilet training issues
		Need to include pain and sleep disorder issues
		Need to include a component on child evaluation
Practicality	Intervention design	Complicated for the facilitator, needs time to prepare
		Some content difficult/complex needs more time to deliver
		Easy for recipients to grasp because of lots of pictures
	Cost	Easy to deliver using local resources
		Easy to absorb into existing community center activities
	Implementation readiness: self-efficacy of prospective facilitators	Easy to deliver to fellow caregivers
		Knowledge of things to share with friends
Adaptation	Adaptation (areas of the intervention needing change/improvement)	Need to find appropriate Chichewa names for CP subtypes
		Need to repackage the content on food types in workshop 4
		Need for introduction of other disabilities in children in Workshop 1

#### 3.1.1 Acceptability

Acceptability was described in terms of intervention characteristics related to the intrinsic appeal of the intervention (perceived advantages and potential disadvantages of the program) and the general level of receptivity of the program by the CBO (implementation climate).

##### 3.1.1.1 Intervention characteristics (advantages)

All participants expressed a positive impression regarding the nature of the intervention package and having fellow caregivers deliver it. The basic themes regarding the perceived advantages of the program were: (i) knowledge of my child; (ii) the program was well organized, (iii) lots of learning and (iv) the value of being taught by fellow caregivers.

Caregivers felt that the program enabled them to know their children better and understand their needs and helpful ways in which they can provide care.

*"Because what I have liked are the new things that we have learnt, of course, I already do most of the therapies. But I really did not know what type of CP my child has, but now I know that there are several subtypes and I know to which group my child belongs and I now know that he is a level 4. And most importantly I know that now I need to do a lot of these and these for him to reach another level of ability. I am so happy!"* Caregiver 04

The professional observers appreciated that the program was well organized and packaged. They felt that the material was delivered in a way that was easy to follow

*"It was good in several ways for example the content was easy for the people to understand it was really, well prepared so that depending on the nature of the participants it made it easy for them to follow and it even made sense to me as a therapist that oh this is what is supposed to be done."* Therapist 01

*“The master trainer was well organized and could do it so well we could tell she is experienced in doing this… and the pictures are good, as we can see the training used a lot of pictures which the caregivers could follow and relate to.”* CBO representative 2

Caregivers also felt that the program provided room for learning new important lessons and practices.

*"For me for all the lessons from Monday to Friday, I have found them to be really good because we did not know anything, we were just living with our children but we did not know what to do with them, how to enjoy the child, how to take care of the child and the type of CP that the child has but now we know the types, the levels of CP (narrates the different types and levels of CP)"* Caregiver 07

The concept of the program being delivered by fellow caregivers was also received positively in several ways. Caregivers felt that they would value being taught by a fellow caregiver who knows what they go through, fellow caregivers were perceived as easily approachable since they share the same language and are not strangers. Expert caregivers were also regarded as a potentially valuable support for therapists and would increase access to therapy for children.

*"Whereas with me, I interact with these people even in the community, we greet and chat. They can ask me anything anywhere…Moreover, for me who has the same problem as them to approach them and give advice on things they can do with their child, they will definitely appreciate this advice and not look down on me."* Caregiver 04

##### 3.1.1.2 Intervention characteristics (potential disadvantages)

Physiotherapists identified two potential disadvantages of the program: (i) that the expert caregivers may not know their limits, and (ii) that they may be looked down upon by their fellow caregivers.

Physiotherapists felt that expert caregivers may start practicing beyond the boundaries of their role in the program. The physiotherapists agreed that this would require a precise explanation of roles and continued supervision by therapists to prevent harm.

*"It is really a problem that can arise. So, to me this problem it's a question of making sure, like you put it earlier that there is supervision from a therapist. And even there should be a clear distinction that this is the therapist and this is the trained facilitator, who has been taught to assist the therapists…So, there should be a balance. Frequent checks and balances."* Physiotherapist 02

The physiotherapists also felt that there was the possibility of caregivers looking down on the expert caregivers and slighting their role and work. On this aspect, both the caregivers and therapists acknowledged this possibility and identified “exchange center visits” as a potential solution alongside good attitude and cordial presentation of the expert caregivers toward their fellows, including personal resilience.

*"One disadvantage that I see … what am trying to say is after they get this training won't they pose in the village and say am the therapist? How do we make sure that they should know their position? They should know their position that okay this is our limit if we go further than this, we may harm. So that the only other problem I see should be addressed."* Physiotherapist 01

“We are organized in centers and we have lots of centers. We can arrange that maybe I should go and facilitate at her center (pointing to a fellow caregiver) and she may come and facilitate training at my center as a visiting facilitator. And because they will see that a visitor has come, they will give more attention and be interested to hear what the visitor has brought. Likewise, I can then go to her center and she will support me. I think that could also help in case we feel that we may be looked down upon us” Caregiver 01

##### 3.1.1.3 Implementation climate

Two basic themes arose under implementation climate (i) intervention aligned with CBO vision and goals and (ii) support structures available.

CBO representatives felt that the intervention was well aligned with its goals and vision and hence were optimistic about its success and showed keenness to implement it.

*"This intervention is well aligned to our goals and vision. We have already been making efforts to implement some of these things. Now that we have learnt a lot more, we will implement them better*” CBO representative 01

The committee representatives and physiotherapist from the CBO envisioned how the program would be easily supported by the already existing structure of the community of volunteers. They felt that community volunteers working with the CBO could also support the work of expert caregivers and make things work.

*“In addition, we also have community volunteers across all centers who can support these expert caregivers. We have like 49 of them in total. Each area at least has 3 or 4 volunteers. We encourage these volunteers to visit the homes of children with disabilities monthly and write reports. So, in our organization, this can certainly work."* Physiotherapist 02

#### 3.1.2 Demand

Demand was defined in terms of intervention characteristics related to the extent to which the intervention program was deemed useful and relevant in comparison to existing ones (relative advantages) and the extent to which the program meets the needs of the caregivers of children with CP.

##### 3.1.2.1 The relative advantage of the intervention

Two themes arose related to the relative or comparative advantage of the intervention: (i) a complement to existing interventions and (ii) less burdensome for caregivers.

Participants felt the program would complement the existing physiotherapy services and that it would result in increasing access to frequent therapies therapy for their children.

*"Lessons like these going to our area will be the first time. Although other interventions are going on, we will be adding more and us being the first, we may turn out to be an example for other areas and help spread the program to other areas. And we will be able to do more with our children at home!”* Caregiver 08

*“We tell the caregivers morning afternoon and evening do some therapy but now with the season that we are in for example, they have to go to the farm, they just do not have time. They cannot get time to do therapies. But now they are being encouraged to incorporate therapy into ma activities of daily living of which they would not spend a whole day without doing these. So, I feel this one is feasible considering the setting that the caregivers live.”* Physiotherapist 02

Caregivers felt that the program presented a way of life that would ease their caregiving roles related to implementing therapies at home. The blended approaches presented to them meant that they would not struggle to create extra time for implementing therapies, which they already struggled with.

*“That is what touched my heart A lot because we weren't finding time sometimes but with what you have explained we have known that sometimes as we are doing daily activities like feeding or bathing the child, we can do helpful activities at the same time.”* Caregiver 03

##### 3.1.2.2 Needs met by the program

Caregivers indicated that the program addressed real needs that they had and their perceptions revolved around two basic themes: (i) learning how to deal with practical issues faced and (ii) acquiring new knowledge.

Caregivers appreciated that the program addressed practical caregiving issues such as feeding, positioning and communication, which are things that they had difficulties with.

*“We really appreciate it because such a program as this is needed and needed urgently so that we may assist our fellow caregivers as soon as possible. I can say that all the problems that were identified through the lessons actually exist in our communities, we see them…the feeding problems, positioning our children poorly and even failing to communicate with them! And so, I feel that the program will practically help our fellow caregivers and also improve the lives of many children.”* Caregiver 05.

Caregivers also identified several things that were new to them which they recognized to be very important and useful. Physiotherapists also acknowledged having learnt things that they had never learnt even during their professional training including the management of Cerebral Visual Impairment (CVI)

*"Ah, the needs are there indeed. Coming here we have learnt a lot that we did not know. We have learnt about the different types of CP and levels of function of children with CP and much more which our friends back home do not know."* Caregiver 09

*"Mmmh, in terms of communication I saw that it went well and today as well (referring to the CVI lesson) I also saw that it went well and to say the truth as a therapist I have also learned things that I have never covered before and if we apply them well it can help a lot. So basically, I think this program is useful. That is my overall impression.”* Physiotherapist 02

##### 3.1.2.3 Needs not met by the program

There were other important child-related needs that the professional observers felt were not addressed by the program: (i) toilet training issues (ii) pain and sleep disorders and (iii) child progress evaluation. They recommended the addition of these aspects in this or future programs.

The physiotherapists mentioned how toilet training was one of the issues that caregivers struggled with when taking care of their children.

*“The issue of toilet training is one of the problem areas we are facing in the community centers we offer services. Most of them are very poor, they cannot afford to buy Pampers…You find a 12- or 14-year-old just lying down, a level perhaps 4, not communicating or showing signs whatsoever. They leave such a child at home and find the child in the evening. No one has ever told them about toilet training the whole of the child’s 12 years of life. So, adding a toilet training component would be helpful.”* Physiotherapist 02

The physiotherapists also remarked that pain, sleep disorders and spasticity management could be other areas that caregivers would benefit a lot.

*“But then we could also look at issues of pain and spasticity and sleeping disorders. I don’t know how they can be incorporated whether it should be the same expert caregivers or whether that would be giving them too much to handle.”* Physiotherapist 01

The CBO representatives remarked that assessment of the progress of children’s activity and participation was important and would have been good to include in the program.

*“Perhaps the one side that I did not see was the area of evaluation. How can we see that our child with CP is improving or changing? That is what I also discussed with the therapist that these areas were not touched.”* CBO representative 01

#### 3.1.3 Practicality

Practicality was defined in terms of intervention design, cost and implementation readiness. The section that follows outlines the themes under each of these predefined constructs of practicality and the direct quotes representing participant perspectives, respectively.

##### 3.1.3.1 Intervention design

There were 3 basic themes related to intervention design: (i) complicated for facilitator to prepare, needs adequate time (ii) some content complex, needs more time to deliver (iii) easy for recipients to grasp because of lots of pictures.

The professional observers felt that the program was quite complicated to put together and would require time for the facilitator to master

*"Yeah, this time am speaking from the part of the person delivering the training, that it's complicated, and you have to take it from that point that you ought to prepare very seriously otherwise it cannot come out as good as the expert trainer delivered it. If not, well prepared you could end up confusing the participants. So, I was speaking on the part of the one delivering."* Physiotherapist 01

*“Well, with pictures sometimes… someone else, like a caregiver selected to facilitate, they may interpret pictures differently and participants may fail to understand the things. The expert trainer was well organized and could do it so well because she is used to doing this. Perhaps our facilitating caregivers will need notes to guide them and more practice to be able to use the pictures like she did …”* CBO representative 02

Participants observed that some sections of the program were complex and contained some very new concepts that would require more time to understand.

*"Today's lesson (module 3) was long and hard to understand in a short period. It has a lot of parts; wanting to differentiate that if this child is in this type CP this is how can you take care of him and how can we prepare him in other activities… it has a lot of sections that one needs to understand.”* Caregiver 08

*"If the content is spread over do one topic per day. For example, doing communication alone and not combining it with play. Then we would not say that the content is too much. During this week we have covered everything in five just to showcase the program. However, I feel that if it were delivered in 7 days as designed it would be good."* Physiotherapist 02

Participants acknowledged that the training program was easy for learners to follow, especially owing to the many pictures and practical demonstrations that were used to illustrate concepts.

*“All sides were seen to be easy because of the pictures because we were quick to tell that this picture means that so we were quick to differentiate with the words but the pictures we were quick to capture the idea that when we get home and see a child like this at this stage, I will explain to them like this. That is what I have seen.”* Caregiver 08

*“Maybe just to add on that, the content yes was a lot but because of the pictures and the practical demonstrations at least the participants could grasp a lot of things through the pictures and demonstrations. It’s the one part of the program that made the program really good and made it simple for participants to grasp ideas easily.”* Physiotherapist 02

##### 3.1.3.2 Cost

There were two themes related to cost: (i) easy to deliver using local resources (ii) expert caregivers may expect something in return.

Apart from the printed pictures, caregivers felt they could be able to source all other materials to deliver the program

*"If we take things from the village people will be quick to accept it. All of these materials we can find and make ourselves…at least except the pictures. If we take our local things, we will give them a picture that they can afford the things that they can manage to give the child and they are the things the child will be eating daily that they can find."* Caregiver 01

There was the worry that expert caregivers may not be willing to work without expecting anything in return. They therefore felt that this may affect sustainability. However, representatives felt that the time-to-time incentivization system that the organization followed could be effective for expert caregivers too. Moreover, the caregivers felt that they would not expect to be paid for sharing information and skills during their usual community center activities.

*“The biggest issue is sustainability, we would want maybe to scale up so that we train more people so that we extend the program, so it is 10 then maybe next we can think of another sample so that the program can grow and reach every caregiver…not many people are willing to do this work without expecting anything in return are few.”* Physiotherapist 01

*“Our system is that although the volunteers are not paid, we still give the people incentives from time to time according to the needs that they get. For example, in times of hunger or famine they are supported…and even during the farming season. So, this is a system whereby they don’t get paid, they know that they have somewhere to go when an emergency arises. But it’s not like it’s a pay but at least they know that being attached to the organization I will be assisted when I have a problem.* CBO representative 02

*“Let me add on the same point, we will be sharing with our friends during our usual community center activities, we already share things when others get training like that nutrition training last time. We have learnt this time, then we will pass on to our friends, and those friends will pass on to others. We will not expect to be paid for sharing with friends in our centers.”* Caregiver 09.

##### 3.1.3.3 Implementation readiness: self-efficacy of prospective facilitators

Caregivers felt that following their participation in the workshops during the week, they could be trained as expert caregivers and deliver the program as facilitators. They expressed this in two ways: (i) the material would make it easy to share with fellow friends and (ii) they understood the knowledge and skills to share with friends.

The practical nature of the program made caregivers feel that they would easily be able to deliver the program to their fellow caregivers.

*"On my side, I would not have problems delivering the training because of the skills that I have received. Also, the materials that were used were really helpful and it's those pictures which aided understanding and also remind you as a facilitator to remember things that you may have missed."* Caregiver 06

The caregivers felt confident of their understanding of the material and, their ability to share the knowledge and skills with their fellow caregivers

*"It will not be difficult to teach them because most of the things we will be delivering are things that we have already covered this week. Moreover, the people we are going to teach are not strangers but women whom we interact with daily so it will not be difficult."* Caregiver 02

#### 3.1.4 Adaptation

Three possible areas for adaptations arose: (i) need to find appropriate Chichewa names for CP sub-types, (ii) need to repackage the content on food types in the workshop 4 and (iii) need to include an introduction to other disabilities in children in workshop 1.

Participants recommended that the CP subtype names have better and consistent local translations that will be practical for use during the program implementation.

*“Yes, we are saying that most parts we understood easily because they were all in Chichewa, the hard parts we found were those names that were in English and they may be difficult for our friends back home because of our literacy levels. Yes, so we would have loved if we had Chichewa translations for those sections too.”* Caregiver 04

*"Let's find good Chichewa names for the CP subtypes so that it is not difficult for people to identify and follow. Even for the pattern names, quad, hemi, Di. Let's also find proper names so that when asked the people should be able to name without problems."* Physiotherapist 01

Caregivers recommended repackaging the content on food groups in workshop four ensure consistency with what the caregivers learn at the local health facilities on nutrition. That is: to teach the six food type groups as is taught in Malawi instead of the three food type groups in the MOC-2-CTP manual.

*"I was worried about the food groups. The way we group them and the way our friends are grouping them is different because we expand those groups into 6 groups and give them names while here, we have been given 3 names. That is where my worry was like how will I explain to my friends for them to understand".* Caregiver 08

Caregivers recommended the inclusion of a section that introduces childhood disabilities before narrowing it down to CP. This is because, in their community center programs, they deal with different types of childhood disabilities not only CP. Therefore, they perceived that adding a generic introduction would be beneficial.

*"But I have one concern about a challenge that we might meet when we go back to our community centers. Because in our centers we have different types of disabilities amongst children … And so, I was thinking that in the beginning, you should have explained to us about the different disabilities that are there and then tell us that this time we are focusing on CP."* Caregiver 01

## 4 Discussion

This study sought to explore the feasibility in terms of perspectives of stakeholders on the acceptability, demand, practicality and adaptation of a caregiver-led training program for caregivers of children with CP in a rural Malawian setting. The MOC2CTP was introduced in the Malawian setting for the first time for potential users to appraise before proceeding with implementation in the community. Our findings demonstrate that the intervention was deemed acceptable and that there is demand for such an intervention in this setting. Participants also felt that it would be practical to deliver the program in their setting but that it required contextual adaptations.

The MOC2CTP program was deemed acceptable by participants due to its practical learning opportunities, well-organized structure, and alignment with the CBO’s vision. Participants felt that the program’s activities were not demanding and would allow them to balance their daily activities in the rural setting. Similar positive perspectives were reported in other skills training interventions for caregivers in LMICs, such as the Juntos in Brazil, and Baby Ubuntu in Uganda (12, 13, 19) where the focus was learning and training through daily activities. In rural settings, interventions that integrate into caregivers’ daily lives and provide peer support are more likely to be accepted. Caregivers prefer interventions that support their natural role and help them cope with daily activities rather than focusing solely on technical skills transfer.

Moreover, the concept of teaching and learning from peers was perceived as supportive, encouraging, and motivating. Caregivers felt that they would be easily accepted by fellow caregivers because of shared experiences, culture and language. These motivators have been identified in similar programs like in the Juntos in Brazil, where the experiential knowledge of the “expert mother” was highly esteemed by fellow mothers who felt encouraged and motivated (21). The engagement of a caregiver as a program facilitator enhances the provision of a family-centered rehabilitation service. Experiential peer support goes a long way to positively impact caregiver well-being beyond the improvement of practical skills related to caregiving.

However, a concern was raised regarding the risk of the expert caregiver crossing boundaries. Recent reviews on peer-led interventions for caregivers of children with neurodevelopmental conditions have not reported this concern (22, 37). However, in a study done in 2022 to investigate benefits and challenges of peer support programs, organizers were also concerned about intervention fidelity by “peer supporters” (38). They ensured careful selection of the “peer supporters,” adequate description of their roles and limits and continuous support. Clear definition of boundaries during the training of facilitators and continued supervision have been highlighted as key to the successful integration of peer facilitation (14, 22, 38). There is evidence of successful and safe engagement of peer facilitators in skills training programs with high perceptions of acceptability reported. Mothers of children with Zika-related neuro-disability and children at risk of CP safely and successfully co-facilitated programs in the Juntos and Baby Ubuntu programs in Brazil and Uganda, respectively, (9, 17). Therefore, during the subsequent implementation of the MOC2CTP, boundaries need to be clearly explained and emphasized in addition to the provision of supportive monitoring and supervision.

All participants recognized this intervention as a great need and relevant to fellow caregivers in their setting. Limited access to a single physiotherapist in their area was a major concern, and this intervention was deemed helpful in addressing it. There is a persisting critical shortage of rehabilitation professionals in LMICs, including Malawi (39). Services in most LMICs are limited to urban communities and render rural populations deprived of services (20, 39). Caregiver skills training programs have been found to increase access to services for caregivers of children with CP and other neurodevelopmental conditions. This is mainly because caregiver skills training programs focus on the engagement of non-specialists which enhances access, affordability and availability of services in the community (17, 26). For example, in rural settings of Ghana, South Africa and Uganda, skills training programs like the Getting to Know CP (GTKCP), MOC2CTP and Ubuntu programs respectively, were the most accessible services to caregivers and any other services were hardly accessible (10, 13, 20). It is not surprising that the prospective stakeholders in this study have also recognized that the MOC2CTP would improve services and make rehabilitation more easily accessible.

Moreover, the CBO representatives and caregivers affirmed the practicality of delivering the program in their community. The CBO representatives felt the program could easily be integrated into their existing programs and that incentive system could also support expert caregivers and ensure the continuity of the program. Caregivers were also confident of their ability to deliver the knowledge and skills imparted to them to their fellow caregivers using resources in the community. Stakeholder readiness and the ability of programs to integrate into existing community programs has been identified as a key indicator of feasibility (13). Moreover, in this setting, the existence of community centers and ongoing caregiver support groups presents a platform for ongoing peer support and continued practice. Such a structure is important for sustainability of, unlike settings where organized support groups are not available (18).

On the other hand, the professional observers felt that although the materials and methods were easy for recipients to grasp., they would be quite complex for the expert caregiver to organize and deliver as intended. They recognized that it would take adequate time and exposure to the delivery kit for them to be able to use the teaching materials with their peers. This highlights the importance of adequate training for expert caregivers, which has also been emphasized by various authors on peer-led training (11, 20, 40). The recommendation for this particular training package is 80 h to achieve consolidation of skills. In addition to this, there were modules which caregivers felt that would require more time for their fellow caregivers to adequately understand, which was attributed to low literacy levels. Similar recommendations have been made in other caregiver training programs, emphasizing adequate oral discussion and the use of simple visual representations (18). Therefore, giving more time for facilitators to explain concepts using the pictures will be beneficial in the pilot implementation of this program.

The stakeholders recommended adaptations to the program related to language, information packaging and diversity of childhood disabilities at their community centers these modifications would ensure that the information is easier to grasp. For example, they recommended further linguistic translation of the CP subtypes in the local language, Chichewa and the inclusion of an introduction section that differentiates CP from other neuro-disabilities of children who come for service in the community centers. Participants also recommended including a description of the three food groups in the “eating and drinking” session to reflect caregivers’ knowledge of the six food groups taught in health facilities in Malawi (41). This demonstrates the necessity of contextual adaptation of interventions before moving to the implementation phase of the larger study. According to the WHO, skills training programs for caregivers of children with developmental disorders like CP, should have materials that are linguistically appropriate and relatable (40). Therefore, these suggestions will be useful in subsequent adaptation of the training program before it is introduced to the community.

In addition to the areas for possible adaptation, stakeholders identified other needs which the current program did not address. These were toilet training and pain management, which are aspects not addressed by the current MOC2CTP. In a recent Ethiopian study, caregivers also identified toilet training as an area not addressed by the WHO CST (18). The need to address pain in children with CP has also been affirmed by the children, caregivers and clinicians in several studies in recent years (42, 43). In LMICs, addressing these issues is complex due to limited access to specialized interventions and uncoordinated delivery of available interventions which result in missed opportunities (39). While toilet training goals may not be feasible for all children owing to levels of severity it is worth investigating in future studies how the community platform may be utilized to start addressing these more specialized needs in a meaningful way.

Overall, the perspectives of the stakeholders regarding the feasibility of the training program were positive. They identified several areas that needed to be modified and their suggestions were useful for ensuring contextual adaptation of the program. In preparation for the subsequent pilot implementation, the investigators engaged a team including four clinical physiotherapists, two social science linguists, the caregivers, the CBO executive and the Malamulele Onward organization to work on the areas recommended for adaptation. Conceptual translation of the CP subtypes was done through consultation with clinical physiotherapists, linguists and the caregivers involved in this study. Development of an introductory section on childhood disabilities was done in consultation with the program developers, the Malamulele Onward. This also included repackaging the section on food types in the “Eating and Drinking” module. This was done in consultation with the personnel from the community health center and the caregivers themselves. Considerations for slow-pacing the delivery of the content were made. A day was allocated only one module, to give room for extensive discussion of concepts and practice. This highlights the essential role of intervention stakeholders in providing critical perspectives important for the contextual adaption of innovations from other contexts.

## 5 Strength and limitations

This stakeholder engagement study drew from participatory action research ethos which propagates for active engagement of all stakeholders of proposed interventions at all interactive stages of implementation (44, 45). Inviting these groups of stakeholders or potential users, strengthened the study since it provided the opportunity to generate contextual knowledge in a co-creative way (44, 46). Secondly, a multi-methodological design qualitative design was applied (33, 34) with two different data collection methods, namely dyadic interviews and focus group discussions. This method took advantage of the strengths of both, as reported by Creswell (47) and augmented the trustworthiness of this study (32). For example, interviewing professional observers in a dyad enabled the co-generation of ideas between the professionals that would not be possible through separate in an individual In-depth Interviews.

Furthermore, there was triangulation of perspectives from all three sets of data to obtain a better understanding of concepts. For example, physiotherapists felt that a peer facilitator would be looked down upon by fellows, however when the researcher posed this concern to caregivers themselves their perspective was that being looked down upon would be because of the way they would present themselves to their peers. They highlighted the need to remain humble and empathetic to earn respect from their peers. This strengthened the rigor of analysis and interpretation and also complemented the researcher’s reflexivity.

This study had an important limitation. The expert trainer only had 5 days to demonstrate the delivery of the intervention, which comprised seven workshops. It would have been ideal if each workshop and subsequent discussion were held on separate consecutive days rather than delivering seven workshops over 5 days, which meant that some of the workshops were combined into one session. Although the aim of this initial exposure to the program was for stakeholders to merely appreciate how the program is designed and delivered, the short period may have affected the perception of stakeholders. Lastly, the views of the stakeholders in this study may not be generalized to other contexts, however, the process and suggestions may be useful when adopting similar interventions in other settings.

## 6 Conclusion

Caregiver-led and delivered interventions offer an innovative way of supporting caregivers of children with complex disabilities like CP in low-resource settings. The MOC2CTP designed by Malamulele Onward was deemed to be acceptable and needed in a rural setting in Malawi. Several suggestions were also made regarding ways to ensure ease and practical delivery of the intervention in the particular rural context of interest. These aspects of practicality and adaptation are discussed further in a future paper and will be used to improve the design of the program in preparation for its implementation. It is evident that the caregiver training program was found useful and tailored to the needs of the stakeholders. However, the contextual adaptations highlighted are important for the intervention to work optimally. Similar stakeholder involvement is recommended for transferring other innovations which strengthens community ownership and transformative co-development of innovations.

## Data availability statement

The original contributions presented in the study are included in the article/supplementary material, further inquiries can be directed to the corresponding author.

## Ethics statement

The studies involving humans were approved by the Human Research Ethics Committee (Medical), University of Witwatersrand, South Africa and the Kamuzu University of Health Sciences Research Ethics Committee, Malawi. The studies were conducted in accordance with the local legislation and institutional requirements. The participants provided their written informed consent to participate in this study.

## Author contributions

TB: Conceptualization, Formal analysis, Investigation, Methodology, Project administration, Resources, Writing – original draft, Writing – review & editing. GS: Conceptualization, Methodology, Supervision, Validation, Writing – original draft, Writing – review & editing. WS: Conceptualization, Methodology, Supervision, Validation, Writing – original draft, Writing – review & editing.
